# Biomolecular Markers of Recurrent Implantation Failure—A Review

**DOI:** 10.3390/ijms221810082

**Published:** 2021-09-18

**Authors:** Aleksandra E. Mrozikiewicz, Marcin Ożarowski, Piotr Jędrzejczak

**Affiliations:** 1 Department of Infertility and Reproductive Endocrinology, Poznan University of Medical Sciences, Polan 33, 60-535 Poznań, Poland; a.mrozikiewicz@gmail.com (A.E.M.); piotr.jedrzejczak@ump.edu.pl (P.J.); 2 Department of Biotechnology, Institute of Natural Fibres and Medicinal Plants—State Research Institute, Wojska Polskiego 71B, 60-630 Poznań, Poland

**Keywords:** recurrent implantation failure, endometrial receptivity, vaginal microbiota, risk factors

## Abstract

Currently, infertility affects 8–12% of reproductive age couples worldwide, a problem that also affects women suffering from recurrent implantation failure (RIF). RIF is a complex condition resulting from many physiological and molecular mechanisms involving dynamic endometrium–blastocyst interaction. The most important are the endometrial receptivity process, decidualization, trophoblast invasion, and blastocyst nesting. Although the exact multifactorial pathogenesis of RIF remains unclear, many studies have suggested the association between hormone level imbalance, disturbances of angiogenic and immunomodulatory factors, certain genetic polymorphisms, and occurrence of RIF. These studies were performed in quite small groups. Additionally, the results are inconsistent between ethnicities. The present review briefly summarizes the importance of factors involved in RIF development that could also serve as diagnostic determinants. Moreover, our review could constitute part of a new platform for discovery of novel diagnostic and therapeutic solutions for RIF.

## 1. Introduction

Recurrent implantation failure (RIF) is defined as three or more consecutive failed in vitro attempts with at least four high-quality embryos in a minimum of three fresh or frozen cycles [1]. This serious complication refers to women younger than 40 [2,3]. It is estimated that 5% of women suffer from recurrent pregnancy loss (RPL), in which 75% of cases are diagnosed as RIF. Among patients under infertility treatment, 15% experience RIF [4].

**Figure 1:**
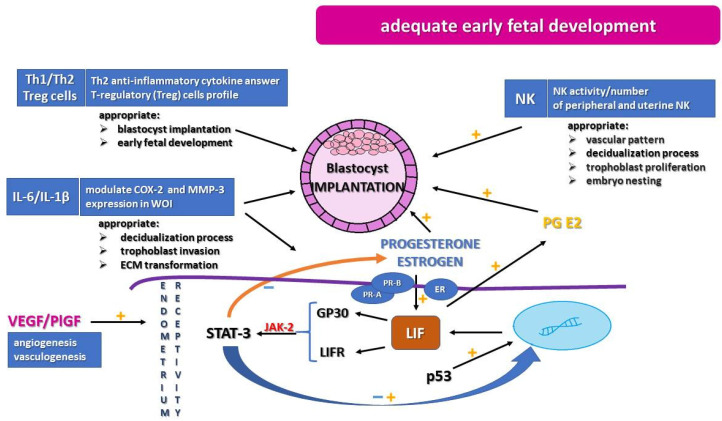
Creation of utero environment for appropriate endometrium receptivity, trophoblast proliferation, and embryo nesting. COX-2, cyclooxygenase-2; ECM, extracellular matrix; ER, estrogen receptors; GP130, glycoprotein 130; IL-1β, interleukin-1β; IL-6, interleukin-6; JAK-2, Janus kinase 2; LIF, leukemia inhibitor factor; LIFR, membrane LIF receptor; MMP-3, metalloproteinase-3; NK, natural killer; p53, tumor protein p53; PlGF, placental growth factor; PR-A/PR-B, progesterone receptors; STAT-3, signal transducer and activator of transcription 3; Th1/Th2, T cells involved in inflammatory response; Treg, T-regulatory cell profile; WOI, window of implantation; VEGF, vascular endothelial growth factor.

A successful pregnancy is a result of proper embryo implantation, followed by adequate decidualization, as well as placentation [2]. Each step requires many molecular and physiological processes for making it possible to achieve the balance necessary for pregnancy development. Implantation is a combination of proper endometrial function and dynamic interaction between the endometrium and the blastocyst. The most important is the implantation phase, which is a multipart process and requires stability in adhesion of the blastocyst, invasion of trophoblast cells, and immune modulation. Achieving implantation strongly depends on proper structure and adequate receptivity of the endometrium (Figure 1). The specific time of the higher rate of successful implantation, called the window of implantation (WOI) (days 20–24 of the cycle, during the secretory phase), depends on high receptivity of the endometrium. Endometrial receptivity is noted upon the appearance of pinopodes, which are microvilli on the apical surface of the epithelial cells (days 20 and 21 of the cycle, up to 5 days of variation). However, without a doubt, endometrial receptivity is a result of many physiological and molecular mechanisms. During the in vitro process and embryo transfer procedure, it is essential to recognize WOI and to maintain endometrial receptivity. Growing evidence suggests that performing elective frozen or thawed embryo transfer may enhance proper implantation (“freeze-all policy”) [5].

Implantation failure is diagnosed as a lack of ultrasound signs of pregnancy in the uterine cavity. In several studies, a biochemical pregnancy was included (an increase in β-hCG without any ultrasound sign of pregnancy) to the definition of RIF. Because the implantation process is complex, the assessment of causes of RIF should be performed on several levels. The most common analyses are chromosomal testing of both parents, the estimation of ovarian function (FSH, LH, AMH measurement) in women, and sperm DNA fragmentation in men, as well as assessment of uterine pathologies and fallopian tube permeability (hysterosalpingogram, laparoscopy) [6,7].

The known risk factors of RIF include childbearing age, BMI, tobacco and alcohol intake, and history of endometriosis. The reasons for RIF could be also divided into embryo and uterine factors. On the other hand, the influence of the male and female factors on the occurrence of RIF has also been shown (Table 1). Although the exact multifactorial pathogenesis of RIF remains unclear, the current review tries to briefly summarize the factors involved in RIF development.

## 2. Immunological Profile

The proper immune response plays a crucial role in receptive endometrium during the WOI [34]. A precise immunologic balance at the maternal–fetal interface in the endometrium is needed to obtain a positive pregnancy outcome. In the early stage of pregnancy, the stability at the maternal–fetal surface is complex and involves immunological processes, including activity of innate lymphocytes, macrophages, decidual dendritic cells, and T cells. All of these cells play a pivotal role in immune acceptance of the embryo, and hence they are also involved in adverse pregnancy outcomes such as the RIF pathomechanism [35]. Innate lymphoid cells (ILCs) were identified in human decidua as playing an important role in maternal–fetal interface immunity and were classified into two subsets: natural killer (NK) cells and non-cytotoxic helper ILCs (ILC1s, ILC2s, ILC3s). The uterine dendritic cells play a vital role in the subtle balance in the decidual space. They are involved in angiogenesis and endometrial stromal cell proliferation and differentiation, as well as in controlling the immune response via T cell-mediated effects [36]. Macrophages are important cells regulating the microenvironment of the developing embryo and are involved in implantation, placentation, and embryo development. These cells are also linked with vascular remodeling at the maternal–fetal interface via promoting the invasion of trophoblasts into spiral arteries. Additionally, they remove apoptotic cells and cellular debris [37]. In the decidua, macrophages are divided into M1 (classically activated) and M2 (alternatively activated) cells. In the luteal phase and physiological healthy pregnancy, M2 macrophages are present in high numbers in the endometrium [37]. It is a crucial to maintain the correct proportion of M1/M2 cells for the appropriate pregnancy outcome; thus, a disproportion in the M1/M2 macrophage ratio leads to several complications during pregnancy, such as preeclampsia, intrauterine growth restriction, spontaneous and recurrent abortion, and RIF [37,38,39].

Many studies have indicated the significant role of dysregulation in the cellular immune response, including imbalance in natural killer (NK) and T cells in receptive endometrium development (Figure 1). This imbalance manifests as intensification of NK activity and augmentation of peripheral and uterine NK cell numbers, indicating the role in RIF etiology [10]. Apart from a uterine presence, NK cells exist in three subpopulations with different immunological features, and separation of the peripheral and uterine NK cells is difficult, so the activity of NK cells should be considered carefully. The abnormal activity of uterine NK cells could be a cause of disturbances in vascular pattern, ischemic symptoms, and augmentation of oxidative stress, which is very unfavorable in the early stage of trophoblast invasion [9,40]. On the other hand, it is recognized that the Th2 anti-inflammatory cytokine response, such as the T-regulatory (Treg) cell profile, supports appropriate implantation and early fetal development. Disturbances in Th1/Th2 profile are associated with recurrent miscarriages, and increased Th1 cytokine pro-inflammatory profile and Th17 phenotype are implicated in recurrent implantation failure [41].

All these pathological processes could lead to the creation of a cytotoxic environment in utero, with inappropriate endometrium receptivity, difficult trophoblast proliferation, and impossibility of embryo nesting. Considering the above-mentioned facts, many studies focus on the inflammatory immune response, which probably plays a vital role in RIF etiology. It was revealed that several cytokines could be factors predicting the occurrence of RIF [7].

Interleukins, such as interleukin-6 (IL-6), leukemia inhibitor factor (LIF), and IL-1 cytokines, play an important role in embryo development and the occurrence of the receptivity window. IL-6, which is synthesized by macrophages, fibroblasts, epithelial cells, and placental trophoblasts, is a multifunctional cytokine greatly contributing to fertility process as well as to the acute immune response. IL-6 level is observed to be high in the luteal phase, particularly in the receptivity window. It is involved in placenta and pregnancy development [42,43]. IL-1a, IL-1b, and the receptor antagonist IL-Ira belong to the same family, have similar biological effects, and are involved in the immune response. They act by the same receptor: IL-1 receptor (IL-1R). These cytokines are shown to be very important in modulation of the process of maternal endometrium decidual change and embryo development. IL-1b, expressed in a high level in the first trimester of pregnancy, is secreted by cytotrophoblast cells. Importantly, IL-1b modulates the expression of cyclooxygenase-2 (COX-2) and metalloproteinase-3 (MMP-3), both recognized to be crucial in homeostasis of decidualization, trophoblast invasion, and extracellular matrix (ECM) transformation [44].

The significant element of the endometrium during the WOI period is the pinopodes, considered to have an important role in endometrial receptivity. Qiong et al. evaluated the clinical value of pinopodes in humans. They concluded that pinopode scoring is a reliable marker of endometrial receptivity and could predict successful embryo transfer [45].

## 3. Leukemia Inhibitor Factor

One of the most important factors modulating endometrium receptivity is leukemia inhibitor factor (LIF), a pleiotropic cytokine belonging to the IL-6 superfamily and known to play a major role in a wide array of physiological processes. During the receptivity window, LIF promoting the development of pinopodes supports blastocyst attachment and invasion and the decidualization process [46].

Several studies have shown the decreasing level of LIF in women with recurrent implantation failure. In the study of Stewart et al., knockout mice deficient in the LIF gene were fertile and were able to conceive and develop a blastocyst, but implantation did not occur [47]. Song et al. suggest that reduction in prostaglandin synthesis may influence the implantation failure connected with LIF alteration [11]. LIF provides upregulation of prostaglandin E2 and stimulates trophoblast development. Prostaglandins are mediators of decidualization [48]. Implantation may be induced after administration of exogenous LIF; hence, subcutaneous injection of LIF may increase the success rate of implantation [12,49]. Another association between LIF and implantation was found in the research on emergency contraception. One of the properties of levonorgestrel is to inhibit LIF, which is a suggested contraceptive mechanism [50]. LIF regulates cellular function via binding to the membrane receptor LIF receptor (LIFR) and glycoprotein 130 (GP130) and activates the downstream signal transduction pathway as a signal transducer and activator of transcription (STAT) [13]. After binding, the receptors dimerize and recruit the kinase Janus kinase 2 (JAK-2), which activates signal transducer and activator of transcription 3 (STAT-3). STAT-3 acts as a gene regulator in the nucleus, and activation of STAT-3 by LIF coincides with the beginning of the WOI [51]. A study of Hiraoka et al. addressed the role of STAT-3 and showed that uterine and stromal STAT-3 may have a crucial role in the implantation process and may suppress the estrogenic response and endometrial proliferation. Thus, it indicates embryo attachment [52].

LIF expression could be altered by estrogen and progesterone. In a mouse model, estrogen can significantly increase expression of LIF in the endometrium during the WOI [49]. Choi et al. observed an alteration in progesterone/estradiol and the LIF pathway. Although changes in expression of hormonal receptors were not observed, decreases in expression of STAT-3 and a gene set associated with the JAK–STAT pathway were demonstrated in patients with RIF. Downregulation of 22 genes metabolizing estrogen and progesterone in the endometrium was detected [53]. Sun et al. observed an elevated estrogenic response in the endometrium connected with STAT-3 gene deficiency [54]. STAT-3 knockout mice demonstrated upregulation of an estrogen-responsive gene and downregulation of some progesterone-responsive genes, which highlighted the reduction in P4-signaling pathways in the endometrium [54,55]. Additionally, LIF is a gene target for p53, which increases its expression. The molecule p53 binds to the p53-binding element in the first intron and alters the expression of LIF in different tissue, including endometrial tissue. The absence of p53 leads to the reduction in LIF and impairment of the implantation process [49]. Considering pleiotropic LIF function, it remains clear that this factor is recognized as a clinical marker of endometrial receptivity occurrence.

## 4. Glycodelin-A

Glycodelin-A (GdA), an abundant secretory glycoprotein in the first-trimester decidua, is one of the important molecules taking part in feto-maternal tolerance, as well as in placental development in early pregnancy. GdA is a progesterone-induced glycoprotein secreted by endometrial glands into the uterus cavity. GdA, dominant in mid-secretory phase, occurs in six different glycosylated glycoforms, which differ in expression in each phase of the menstrual cycle. The presence of GdA in endometrial epithelial and stromal cells has been observed [15].

During proper implantation, GdA stimulates an endometrial proliferation and regulates an attachment of trophoblast and affects cell proliferation, differentiation, adhesion, and motility. Therefore, GdA is a vital molecule during trophoblast and placenta development [16,56]. There is also evidence that GdA could play a major role during implantation, modulating numerous processes, especially the immunosuppression mechanism. In this field, GdA reduces the cytotoxic effect of NK cells and modulates NK cells to cytokine production, with a balance shift to Th2 cytokines [57,58,59]. Moreover, GdA inhibits T cells proliferation, as well as stimulates T cells apoptosis and modulates the immune answer from B cells [17,60]. The important role of GdA in modulating the suitable phenotype of dendritic cells has also been shown [61].

GdA level could indicated to appropriate endometrial receptivity and the chance for successful implantation. It was revealed that GdA, as well as mucin-1 (MUC-1) in the human endometrium and in serum blood during WOI period, were significantly lower in RIF women compared with fertile women. Additionally, the correlation of blood and tissue levels of MUC-1 and GdA has been observed. [62]. These remarks indicate that the changed expression profile of GdA could be associated with impaired endometrial receptivity and could play an important role in recurrent implantation failure, early miscarriage, and the recurrent miscarriage pathomechanism [15,16]. Considering the above-mentioned findings, the GdA could be a good potential biomarker of endometrial activity.

## 5. Progesterone, Estrogen, and Their Hormonal Receptors

Extensive studies have demonstrated the important role of progesterone, estrogen, and hormonal receptors during implantation. Physiologically, after ovulation the estrogen level decreases and the progesterone level increases. Increased levels of estrogen and LIF in endometrium coincide with implantation time [49].

The correct balance between estrogen and progesterone enhances proper endometrial proliferation during the WOI and prepares the endometrium for blastocyst invasion. In women with RIF, the implantation could be hampered by an elevated estradiol level during ovarian stimulation that could lead to premature rising progesterone levels and maturation of the endometrium [49]. Several studies focus on this significant problem. Prapas et al. suggest that the time of endometrial exposure to a high progesterone level may distinguish the WOI during the menstrual cycle [63]. Klonos et al. assessed expression of estrogen (ER) and progesterone receptors (PR-A, PR-B), comparing receptor expression in the endometria of healthy women undergoing an oocyte donation program on days 0 and 5 of oocyte retrieval. A statistically significant increase in expression of PR-B and decrease in ER were observed, confirming the physiological changes in the endometrium during preparation for implantation. The expression of PR-A remained constant [64].

PR-B gene knockout mice had a reduced implantation rate in comparison with PR-A knockout mice, suggesting the relevance of PR-B expression for implantation [65]. In patients suffering from RIF, a decrease in ER was observed [66]. Similar results were obtained in another study, which showed a statistically significant correlation between ESR1 polymorphism and implantation failure [67].

Several authors suggest crucial interaction between p53, LIF, and hormonal receptors in the reproduction process. The multi-functional protein that is the p53 molecule plays a crucial role in several physiological processes, such as genomic stability, tumor suppression, and gene expression regulation. In numerous studies, its role in the reproductive system was demonstrated. Through inducing apoptosis and angiogenesis, it influences endometrial receptivity, as well as oocyte maturation and quality [68]. The p53 impact on implantation is meditated by LIF [49]. Furthermore, VEGF is one of the gene targets of p53, and alteration in p53 expression leads to an increase in VEGF tissue expression through hypoxia-induced factor [69].

## 6. Angiogenic Factors

Vascular endothelial growth factor (VEGF) is an angiogenic factor with a pleiotropic role in numerous pathophysiological processes. The VEGF family includes several proteins (VEGF-A to VEGF-F), as well as placental growth factor (PlGF), among which VEGF-A is recognized as the most important factor of angiogenesis involved in the regulation of endothelial cell function. It also seems that VEGF-A plays a crucial role in embryo implantation and placenta development during the early stage of pregnancy. In addition, placental expression of VEGF-A increases endometrial receptivity and positively improves the interactions between the embryo and endometrial tissue. VEGF also dynamically acts in angiogenesis and vascularization of the embryo. It was demonstrated that proper VEGF and PlGF gene expression is vital for embryo implantation, trophoblast invasion, adequate angiogenesis, and placenta development in successful pregnancy [70] (Figure 1). Both progesterone and estrogen are regulators of VEGF synthesis [71]. That means that embryo implantation is conditioned by estrogen, progesterone, and angiogenetic factors and that all of these should be taken into account as potential factors in this mechanism.

Moreover, both VEGF and PlGF could also modulate the immuno-tolerance processes in the maternal immune system throughout the embryo implantation, activating the monocytes and macrophages [72]. In addition, PlGF promotes the proliferation and differentiation of uterine NK cells, as well as dendritic cells, and could be able to balance towards the Th2 phenotype. The above-mentioned immunoregulatory roles of VEGF and PlGF are crucial for the initiation and development of pregnancy [73].

Endometrial VEGF expression is reduced in the peri-implantation period in infertile women [74], as well as in women with recurrent miscarriages [75]. In patients with RIF, an elevated level of VEGF in blood and a decreased level of endometrial expression are observed. The results show the significantly lower level of MMP-7 and VEGF in peripheral blood in the RIF women compared with the controls, reflecting the role of these circulating molecules in the implantation process. Both of these molecules could be proposed as biomarkers of RIF development [76]. In several studies, altered expression of VEGF in recurrent miscarriages shows the importance of the angiogenic factor in placenta development [77,78].

The main functions of PlGF, another important member of the VEGF family, are chemotactic, mitogenic, and angiogenic activity of endothelial cells. On the other hand, PlGF also has chemotactic function for monocytes and several other hemopoietic cells. This molecule is expressed during embryo implantation by maternal uterine NK (uNK) and fetal trophoblast cells. PlGF is reported to be associated with pathological angiogenesis, which occurs in inflammatory processes and tumor angiogenesis [79]. The decreasing concentration of PlGF in preeclamptic women suggests that this molecule could predict occurrence of the disease [80,81]. It was suggested that PlGF could play an important role in uterine NK cell proliferation, as well as in differentiation.

Because PlGF and VEGF act by the same VEGF receptor (VEGFR-1, fms-like tyrosine kinase-1), they each potentiate the biological effects of the other. PlGF and VEGF are expressed by maternal endometrial cells, and in addition, PlGF is abundantly expressed in the trophoblast and placenta (villous cytotrophoblasts, syncytiotrophoblasts, extravillous trophoblasts, villous mesenchyme). Both PlGF and VEGF are expressed by uterine NK, endometrial glands, and macrophages [74,82].

The significant role of uterine NK in pregnancy is to modulate the angiogenesis and vasculogenesis in the early stage of trophoblast invasion [73].

## 7. Genetic Factors

Various genetic factors are involved in the critical process of embryo implantation, and several single-nucleotide polymorphisms (SNPs) have been reported to be associated with RIF.

MicroRNAs, recognized function modulators, could control the expression of many genes involved in the peri-implantation period and in this way could play a vital role in fetal/placental development. On the other hand, these factors might be closely involved in recurrent implantation failure (IVF) and recurrent pregnancy loss (RPL) pathogenesis [28].

In the population of Korean women, the significant role of miR-449bA>G polymorphism (AG + GG genotype was associated with RIF prevalence, OR = 1.584, *p* = 0.046) [3] as well as miR-27a rs895819 and miR-449b rs10061133 [83] in RIF predisposition and RPL development was observed. Despite these positive observations, the exact mechanism underlying the function of microRNAs in RIF etiopathology remains unclear [3].

In several analyses it was shown that inherited thrombophilia could be a risk factor of recurrent RIF failure. Safdarian et al. observed that the presence of factor V Leiden mutation (OR = 3.06, *p* = 0.01) and the homozygote form of methylene tetrahydrofolate reductase (MTHFR) mutation (OR = 12.33, *p* = 0.05) were significant risk factors for recurrent IVF failure [27]. In addition, the occurrence of at least one thrombophilia was a risk factor of recurrent IVF failure (OR = 3.15, *p* = 0.00). In the study performed by Qublan et al., at least one inherited or acquired thrombophilia was determined in 68.9% of the RIF group vs. 25.6% in the group with successful pregnancy after the first IVF–embryo transfer cycle and 25% in the control group, which included women with uneventful pregnancy and without miscarriages in their history (*p* < 0.01). Combined thrombophilia was more frequent in the repeated IVF failure group vs. the two above-mentioned control groups (35.6% vs. 4.4% and 3%) (*p* < 0.0001). The authors demonstrated the crucial role of acquired and inherited thrombophilia in IVF–embryo transfer implantation failure and concluded that women with repeated IVF–embryo transfer failure should be screened for thrombophilia [84]. In contrast, several analyses did not reveal a significant role of thrombophilic factors in IVF–embryo transfer implantation failure [85,86,87]. In a meta-analysis performed by Zeng et al. (nine studies including 1812 women: 754 RIF vs. 1058 controls), no association between either MTHFR polymorphism (MTHFR C677T or MTHFR A1298C) and RIF occurrence was confirmed [88].

Several studies have revealed the possible role of p53 genetic polymorphisms in RIF occurrence. An SNP in codon 72, replacing G with C, which results in proline (P72) instead of arginine (R72) in the protein chain, was identified as modulating the biological activity of protein p53, consequently influencing the reproductive capacity [30,87,88]. Interestingly, the women carriers of the C allele (proline) of p53 codon 72 polymorphism showed decreased leukemia inhibitory factor expression, thus leading to a lower rate of implantation success [14].

An earlier study by Kang et al. showed that p53 P72 allele presence was higher in women undergoing in vitro treatment in comparison with the general population (33.1% vs. 22.7%, respectively, *p* < 0.005). Additionally, this polymorphic variant identified in women younger than 35 appears to be a risk factor for implantation failure (implantation rate 19% vs. 42% in homozygous and heterozygous for P72 allele, respectively, *p* = 0.0028) [89]. These findings were confirmed in an analysis conducted by Lledo et al., who indicated that R72P polymorphism of the p53 gene is more frequent in the group of RIF and RPL patients compared with the fertile population [90]. In addition, it was also suggested that rs1042522 (R72P, G/C) and rs17878362 (Ins16bp, N/D) variants of the p53 gene could be a genetic factor predisposing to RIF [91,92].

Human leukocyte antigen (HLA)-G is recognized as an important factor of the immunomodulatory system, modulating the fertilization process as well as influencing the early stage of pregnancy; thus, its expression has been the subject of numerous analyses in women with RIF. Several studies have shown that polymorphisms of the HLA-G gene could be involved in effective implantation after the IVF–ETs technique. The significant role of several HLA-G polymorphism (rs1632947, rs1233334, rs371194629 HLA-G) in infertile patients, such as soluble HLA-G in the early stages of pregnancy, was indicated in the study of Nowak et al. in a population of Polish women [31]. On the other hand, in a large meta-analysis, Fan et al. found that an HLA-G 14-bp insertion allele probably increases the risk of RIF in Caucasians. In a meta-analysis, the authors found that HLA-G 14 bp polymorphism was significantly correlated with RIF occurrence (OR = 1.74) [93].

The significant role of angiogenetic factors, especially VEGF, in the implantation process is not in doubt. In a metanalysis, in a group of 683 patients (305 RIF women, 378 controls), the authors observed the strongest association between the −1154A allele of the VEGF gene and RIF occurrence (OR 1.39, *p* = 0.01). These results indicated that the –1154A>G variant may be perceived as a risk factor of RIF [54]. Additionally, other studies showed that women carrying several genetic variants of VEGF genes would be at significant risk of RIF occurrence [92].

Additional factors associated with the implantation process are several variants of the **estrogen receptor 1** (ESR1) gene. Vagnini et al. investigated the role of the ESR1/AA (rs12199722) genotype, which was common in the RIF group compared with women who became pregnant on their first cycle of IVF/intracytoplasmic sperm injection (OR = 7.9), as well as compared with women who became pregnant without treatment (OR = 2.8) [67]. This observation reveals the important role of this genetic polymorphism in RIF etiology.

## 8. Vaginal and Endometrial Microbiome (Microbiota) Disturbances

Many studies have shown that *Lactobacillus* species (mainly *L. crispatus*, *L. gasseri*, *L. iners*, *L. jensenii*), as the dominant component of the vaginal microbiota, can play a protective role and may possess a eubiotic effect on the vaginal microenvironment [77,94,95]. It is known that *Lactobacillus* species inhibit invasion and colonization of pathogenic bacteria by production of a high concentration of lactic acid and short-chain fatty acids, which can maintain the acidic and anaerobic environment in the vaginal area [18,96] and which possess antibacterial, antiviral and immunomodulatory properties [97]. Vaginal lactic acid may disrupt the outer membrane of Gram-negative bacteria such as *Pseudomonas aeruginosa* and *Escherichia coli* [98,99,100]; it can inhibit opportunistic infections caused by *Gardnerella vaginalis*, *Trichomonas vaginalis*, *Neisseria gonorrhoeae*, *Chlamydia trachomatis*, herpes simplex virus (HSV), and human *Papillomavirus* (HPV) [101]. Moreover, *Lactobacillus* species can produce antimicrobial peptides (bacteriocins, bacteriocin-like substances) and biosurfactants [68]. According to the latest data, it is estimated that the vaginal microbiota contains approximately 10^10^–10^11^ bacteria, whereas the endometrium harbors 4 orders of magnitude fewer bacteria than the vagina [19,77].

It should be emphasized that the vagino–uterine microbiota and its dysbiosis may influence the pathogenesis of several gynecological infectious and non-infectious illnesses [18,20,102], including gynecologic cancers [103]. Recently, it was observed that dysbiosis of the vaginal microbiome may be involved in recurrent implantation failure (RIF) at various stages, such as formation of gametes, implantation, and delivery [18,104]. Moreover, Moreno et al. [105] observed that the microbiota in the endometrial fluid is mainly dominated by *Lactobacillus* spp. (>90%), in comparison with *Gardnerella*, *Streptococcus*, and *Bifidobacterium*. However, in another group of women the endometrial microbiota was dominated by bacteria other than *Lactobacillus* spp., such as *Atopobium*, *Bifidobacterium*, *Gardnerella*, *Megasphaera*, *Prevotella*, *Sneathia*, and *Streptococcus* [19]. This kind of endometrial microbiota, along with a low level of *Lactobacillus* spp., has been associated with negative reproductive outcomes in patients—women had significantly lower implantation, pregnancy, ongoing pregnancy, and live birth rates, as well as higher miscarriage rates [105]. Another study [19] showed that endometrial dysbiosis may cause infertility. Although the endometrial and vaginal microbial populations are different, the key to recurrent implantation failure is *Lactobacillus* spp. It is well known that uterine infections caused by *Streptococcus* spp., *Staphylococcus* spp., *Enterococcus* spp., *Escherichia coli*, and *Klebsiella pneumoniae* are risk factors of infertility because inflammation in the reproductive tract may lead to impairment of embryo implantation and the onset of a successful pregnancy [19]. According to Schoenmakers and Laven [96], the relative abundance of *Lactobacillus crispatus* (>60%) and *Lactobacillus iners* (>60%) in the vagina may be used for the classification of women with higher chances to achieve a pregnancy during assisted reproductive technology. A recent review of studies [106] confirmed that *Lactobacillus* abundance in the female genital tract is associated with better reproductive outcomes and results of assisted reproductive technology. All risks factors are summarized in Table 1.

## 9. Treatment of RIF

It is worth underlining that the identification of RIF risk factors could also be useful in clinical practice. There is growing evidence that immunological therapeutic methods have an effective role in RIF patients. Ahmadi et al. revealed the benefits of using intravenous immunoglobulin (IVIG) therapy in RIF women. The authors evaluated 72 women with RIF who were divided into two subgroups: women who received IVIG, aspirin, and heparin (anoxaparin) and women who received aspirin and heparin (anoxaparin) and no IVIG. In all women, the function of Th17 and Treg cells, as two subgroups of CD_4_^+^ T cells, in implantation and pregnancy rates were assessed. It was found that the use of IVIG affects the regulation of immune mechanisms, especially Treg cytokines, and in this way could be an optimal choice in the treatment of implantation failure [107]. Furthermore, for the improvement of pregnancy rate, lymphocyte immunotherapy (LIT) and IVIG have been applied. In combination, they might augment the efficacy of embryo implantation through several processes, such as modulating Th1/Th2 balance, increasing T regulatory cells (Tregs), and inhibiting NK activity [107,108]. The benefits of using immunomodulatory treatment before embryo transfer were demonstrated in an interesting study by Kolanska et al. In RIF women, steroids and intralipid treatment were used. In this group, clinical pregnancies occurred significantly more frequently in treated versus untreated embryo transfers (*p* < 0.001). Both steroids and intralipids resulted in higher clinical pregnancy rates [109]. In the other study in women after IVF with a history of recurrent pregnancy loss or recurrent implantation failure, it was also indicated that immunomodulatory (prednisone, intravenous immunoglobulin G (IVIG)) and anticoagulation treatment (low molecular weight heparin and low dose aspirin) suggestively improved the pregnancy [108].

It has also been demonstrated that daily administration of hydroxychloroquine (HCQ) in RIF women downregulated Th17 cytokines and additionally upregulated Treg cytokines. Both of these processes, through influencing the Th17/Treg ratio, compensate for the immune response [110].

## 10. Materials and Methods

In this review, publications available in PubMed and Scopus databases as well as in Google Scholar were taken into account. The following keywords and their combinations were used: “recurrent implantation failure”, “endometrial receptivity”, “vaginal microbiota”, “risk factors”. Additional searches included references from identified publications. In the screening process, articles published in predatory journals and studies published earlier than 1980 were excluded.

## 11. Conclusions

Summarizing the pathogenesis of RIF, the most important roles are played by embryo and uterine factors and disturbances of the hormonal and cytokines network, along with angiogenic and immunomodulatory factors, as well as several multi-functional proteins, such as the p53 molecule. Other factors include the vaginal microbiome, as well as a genetic polymorphism of the gene involved in embryo implantation, immune response, and endometrial receptivity (Table 2).

Recurrent implantation failure is an important problem and a great challenge for human reproductive medicine. Implantation disorders affect the physical and mental health of women and are a common cause of disappointment in both partners, as well as constituting a great difficulty for clinicians. Undoubtedly, identifying the factors predisposing the occurrence of RIF will make it possible to individualize treatment plans and consider the “freeze-all policy” during in vitro treatment, which altogether could increase the rate of implantation success. Studies of RIF biomarkers could improve the chance of pregnancy. Many studies have revealed the association between hormones, angiogenic and immunomodulatory factors, as well as genetic polymorphisms and RIF occurrence, but these observations require further research on large samples of different ethnic populations.

Currently, it is a fact that infertility affects 8–12% of couples in reproductive age worldwide, and it is a growing problem. There is clearly a great need for progress in the development of diagnostic tests that would allow the assessment of RIF and RPL risk.

## Figures and Tables

**Table 1:** The frequent risk factors of RIF.

Risk Factors of RIF		References
Uterine	anatomical abnormalities (bicornal uterus, uterine septa, myomas, endometrial polyps, intrauterine adhesions)	[8]
	immunological (HLA, NK cells, TK cells)	[9,10]
	biomolecular (p53, LIF)	[11,12,13,14]
	glycodelin-A	[15,16,17]
	infection	[18,19,20]
Embryo	genetic abnormalities	[21,22]
Male	sperm quality	[23,24]
Female	thrombophilia, inherited and acquired	[25,26,27]
	other genetic polymorphisms (miRNA, HLA-G, p53, VEGF)	[28,29,30,31]
	vitamin D deficiency	[32,33]

**Table 2:** Summary of pathophysiological processes in relation to RIF development according to environmental and pathophysiological risk factors.

Risk Factor	Pathophysiological Processes in Relation to RIF Development	References
Environmental Risk Factor		
Maternal age	chromosomal abnormalities—aneuploidy, polyploidy, mosaicism, translocations, inversions/deletions	[111,112]
BMI (>25 kg/m^2^)	disturbances in gonadotropin secretion	[113,114]
Tobacco intake	lower estradiol level during ovarian stimulation disturbances of corpus luteum formation and embryo implantation reduction in oxygen supply to the fetus (carbon monoxide) vasoconstriction and decreased delivery of nutrients to the fetus (nicotine) significantly decreased sperm count	[115,116,117]
Stress	elevated cortisol level (stress hormone)	[118]
**Pathophysiological Risk Factors**		
Th1/Th2 imbalance	elevated number and activity of peripheral and uterine NK cells elevated activity of Th1 cells and elevated levels of cytokines produced by Th1 cells (TNF-α)—inflammatory promoting, trophoblastic growth suppression, thrombotic responses in maternal uterine blood vessels, embryo rejection	[9,41,80]
Infection	chronic endometritis—*Escherichia coli*, *Enterococcus faecalis*, group B *Streptococcus*, *Mycoplasma*, *Chlamydia*	[18,19,77,105]
Inherited thrombophilia	carrying of factor V Leiden mutation, deficiency of MTHFR, PTM and ATIII	[27,84,85,86]
Molecules expression	LIF—decreased level leads to influence the reproductive capacity VEGF—reduced expression in peri-implantation period in RIF PlGF—decreasing concentration in RIF	[46,50,70,74,76]
Genetics factors	microRNAs—function modulators, control the expression of genes involved in peri-implantation period	[28,83]
	factor V Leiden mutation, MTHFR mutation—the role in IVF–embryo transfer and implantation failure	[27,84]
	p53—genetic polymorphisms modulate the biological activity of protein p53, leading to lower rate of implantation success	[14,30,87,88,89,90,91,92,119]
	HLA-G—genetic polymorphisms modulate immune system influencing fertilization process	[14,31]
	VEGF—genetic polymorphisms involved in angiogenesis process, support the successful implantation	[29,92]
	ESR1—genetic polymorphisms associated with the implantation process	[67]

ATIII, antithrombin III; BMI, body mass index; ESR1, estrogen receptor 1; HLA-G, human leukocyte antigen G; LIF, leukemia inhibitor factor; MTHFR, methylene tetrahydrofolate reductase; NK, natural killers cells; p53, protein 53; PlGF, placental growth factor; PTM, prothrombin; TNF-α, tumor necrosis factor α; VEGF, vascular endothelial growth factor.

## Data Availability

The study did not report any data.
